# Supplementary figures and images for: Components of the endocytic and recycling trafficking pathways interfere with the integrity of the Legionella‐containing vacuole
Source: Cell Microbiol. 2020 Mar 17;22(4):e13151. doi: 10.1111/cmi.13151 (PMC7154685; doi:10.1111/cmi.13151)

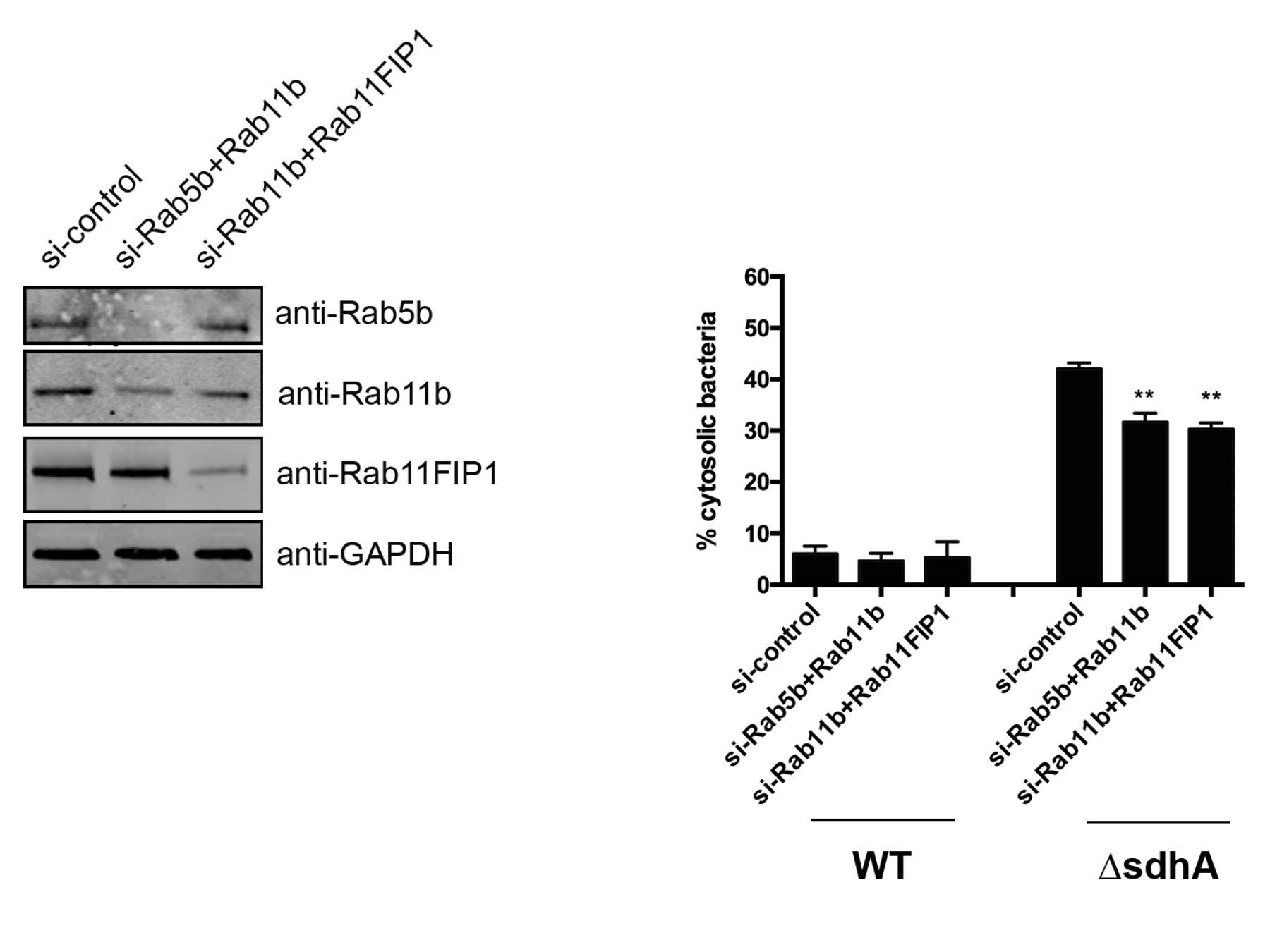

Supplement: Supplementary file 2 — Figure S1. Depletion of Rab11b in combination with either Rab11FIP1 or Rab5b does not further potentiate cytosolic exposure of bacteria. Left: A/J bone marrow‐derived macrophages were nucleofected with noted siRNAs. Knockdown efficiency was assessed by immunoblots with noted antibodies. Right: Nucleofected macrophages were challenged with either WT or ΔsdhA Legionella, fixed at 6 hpi, and immunostained to determine cytosol exposure. Percent of cytosol‐detected bacteria was quantified, as described (Experimental Procedures). [file CMI-22-e13151-s001.tif]
